# Balneotherapy and human immune function in the era of COVID-19

**DOI:** 10.1007/s00484-020-01914-z

**Published:** 2020-04-16

**Authors:** Stefano Masiero, Maria Chiara Maccarone, Giacomo Magro

**Affiliations:** 1 Rehabilitation Unit, Department of Neuroscience, University of Padova, Via Giustiniani 3, 35128 Padova, Italy; 2 Physical Medicine and Rehabilitation School, University of Padova, Padova, Italy

Dear Editor,

In this historical moment (COVID-19 infection spreading worldwide), we think it is interesting to know whether balneotherapy applications as mud-bath therapy or hydrotherapy applications can affect the immune system. Many mineral-rich waters have been shown to have effects on the immune system and recent findings suggest that balneotherapy may improve the efficacy of immune response. The mechanisms through which immersion in mineral-rich water or mud therapy may be useful to improve human immune function are still not completely understood, but surely neuroendocrine and immunological responses, including both innate and adaptive immunity, and also cell-mediated and humoral immunity, are involved.

Concerning cell-mediated immunity, balneotherapy applications can improve the survival capacity and activity of neutrophils. Rinaldi et al. (2006) demonstrated in vitro that sulphurous mineral water favours the short-term survival of neutrophils, speeding up the resolution of infections and preventing further inflammation. Studies conducted in patients with osteoarthritis who underwent hydrotherapy or mud-bath therapy showed that, after the treatment, neutrophils’ circulating and functional capacity increased, reflecting a greater defence capacity against pathogens and thus a potential lower susceptibility to infections (Gálvez et al. 2017, 2018a, 2018b). Balneotherapy may also contribute to increase cortisol levels in healthy and subhealthy people. Changes in cortisol levels suggest that mineral baths may modulate the activity of the hypothalamic-pituitary-adrenal axis, inducing a transitorily but significant rise in ACTH production (Antonelli and Donelli 2018). It is possible to hypothesize that an increase in the systemic concentrations of cortisol after balneotherapy could mediate the stimulation of the phagocytic activity of neutrophils in patients with osteoarthritis (Ortega et al. 2017). Also, radon bath therapy can influence cell-mediated immunity by giving a small but long-lasting increase in monocytes (Rühle et al. 2017).

Regarding the adaptive immunity, immersions in hot baths can increase, probably depending on an increased somatotropic hormone production, the total number and the activity of CD8+ lymphocytes and natural killer cells, important in neutralizing, killing and rejecting cells infected by viruses (Blazícková et al. 2000). On the other hand, Piao et al. (2020) investigated immune function changes in the peripheral blood of people living near radon hot springs, demonstrating an increase in the percentage of CD4+ cells, that induce T and B lymphocytes proliferation, and a reduction in the percentage of CD8+ cells. Balneotherapy can also normalize the proportion of Th1, Th2, Th17 and Treg CD4+ lymphocytes. Th1 cells have a cytotoxic activity against intracellular pathogens (such as viruses, *Mycobacterium tuberculosis*); Th2 cells regulate humoral immunity and allergic responses, whereas the Th17 family is involved in the defence against extracellular pathogens. Th17 cells are pro-inflammatory lymphocytes, while Treg cells have a suppressor activity. A fine balance between Th17 and Treg CD4+ lymphocytes has emerged as a crucial point in the inflammation associated with autoimmune and immune-mediated diseases. Sulphur balneotherapy seems to have a role in the activation of both polarization pathways (Vitale 2018), while in patients who followed bath applications over 3 weeks with low dose radon, only Treg cells were increased after the treatment and Th17 cells were unchanged (Cucu et al. 2017).

Regarding the modulation of inflammatory cytokines’ activity, mud therapy and balneotherapy can cause a reduction in serum concentrations of pro-inflammatory cytokines TNF-α and IL-1β as well as an increase in anti-inflammatory IGF-1 (Gálvez et al. 2018a, 2018b). In a psoriasis-like murine model, balneotherapy suppressed lesional IL-23 and IL-17A, cytokines involved in maintaining chronic inflammation (Lee et al. 2014). C-reactive protein levels, which rise in response to inflammation, decrease significantly after balneotherapy (Gálvez et al. 2018a, 2018b).

In conclusion, it seems that the use of balneotherapy (bath, mud therapy) may improve human immune function, both humoral and cell mediated. Due also to the variety and heterogeneity of balneotherapy modalities, characteristic of applications, water and mud compositions, the possible presence of radioactive properties, it might be interesting to explore this topic. However, we believe that the spas, if hygienically controlled, respecting WHO and national recommendations, can be visited for balneotherapy applications even in these days in which the world is struggling against COVID-19.
